# Application of Δ- and Λ-Isomerism of Octahedral Metal Complexes for Inducing Chiral Nematic Phases

**DOI:** 10.3390/ijms10104559

**Published:** 2009-11-20

**Authors:** Hisako Sato, Akihiko Yamagishi

**Affiliations:** 1 Department of Chemistry, Faculty of Science, Ehime University/Matsuyama, 790-8577, Japan; 2 PRESTO/JST/Chiba, 277-8561, Japan; 3 Department of Chemistry, Faculty of Science, Ehime University, Ochanomizu University/Tokyo, 112-8610, Japan; E-Mail: yamagishi.akihiko@ocha.ac.jp (A.Y.)

**Keywords:** metal complex, chiral, dopant, nematic, twisting power, vibrational circular dichroism

## Abstract

The Δ- and Λ-isomerism of octahedral metal complexes is employed as a source of chirality for inducing chiral nematic phases. By applying a wide range of chiral metal complexes as a dopant, it has been found that tris(β-diketonato)metal(III) complexes exhibit an extremely high value of helical twisting power. The mechanism of induction of the chiral nematic phase is postulated on the basis of a surface chirality model. The strategy for designing an efficient dopant is described, together with the results using a number of examples of Co(III), Cr(III) and Ru(III) complexes with C_2_ symmetry. The development of photo-responsive dopants to achieve the photo-induced structural change of liquid crystal by use of photo-isomerization of chiral metal complexes is also described.

## 1. Introduction

The effect of chirality in liquid crystals has long intrigued scientists in broad disciplines [1–8] since the discovery of a cholesteric liquid crystal in 1888. Molecular chirality continues to be regarded as one of the symmetry lowering elements, particularly in the area of emerging novel phases [7]. There still remains a crucial question as to how the helical ordering is associated with molecular chirality. In the cases of bent–core mesogens, for example, some workers favor a view that molecules of *C*_2v_ symmetry can still give rise to layer chirality [9], if coupled to the tilt, while others seem to believe in molecular conformational chirality implemented in the mesophase. Apart from these fundamental problems, the phenomenon of chiral phase induction has been actively investigated from the practical points in the areas of organic and polymer chemistry, and is recognized as a useful means of elucidating chiral structures on mesoscopic and macroscopic scales [11–13].

Nematic liquid crystal (N) phases are known to transform to chiral nematic liquid crystal (N* or cholesteric liquid crystal) phases on use of small amounts of chiral molecules as chiral dopants [1]. In these phenomena, the chirality of dopant molecules is the cause of induction of a helical structure. In liquid crystal systems, one of the most interesting aspects is that the molecular properties of a dopant molecule are reflected on various levels of molecular architectures. When chiral photochromic molecules are used as a dopant, for example, the helical pitch expands or contracts on a micrometer scale in response to the photo-irradiation [14–23].

The power of inducing an N* phase is measured in terms of Helical Twisting Power (denoted by HTP or *β_M_*) according to the following equation:

(1) $$\beta_{M}={\left(\frac{\partial P^{-1}}{\partial x}\right)}_{x\to 0}$$

in which *x* is the molar fraction of a dopant and *p* the pitch length of an induced helix, respectively [1]. The basic mechanisms of the transition from N to N* phases by chiral molecules have been studied theoretically to predict the HTP on the basis of the surface chirality model [10–12]. In some cases, however, correlation between the structure of a chiral dopant and induced helix is implicit and the magnitude of *β_M_* value, even its sign, is difficult to predict.

A wide variety of organic molecules have been applied as chiral dopants in order to clarify the above problems [3]. In contrast, inorganic compounds are not used so extensively, although the chirality of metal complexes is a main issue in coordination chemistry [24–27]. Motivated by these circumstances, more than ten years ago we initiated an attempt to develop metal complexes as efficient chiral dopants. In this review, we summarize our attempts at using the ΔΛ chirality of octahedral Co(III), Cr(III) and Ru(III) metal complexes in nematic liquid crystals [24–31]. In comparison to the chirality based on asymmetric carbon atoms, the ΔΛ isomerism of metal complexes is differentiated by the following characteristics:

1. The ΔΛ chirality has a more rigid nature than that of asymmetric carbon. Besides its size extends over 1 nm as a whole including a central metal ion and ligands. The scope under the influence of a chiral metal complex is even larger when it includes a bulky planar ligand such as aromatic rings.
2. The molecular properties of a dopant can be changed systematically by changing the coordination structures such as the degree of ligand replacement, geometrical isomers and the linking of coordination units by bridging ligands. This character would be helpful to reveal the structure-function relations on this topic.
3. Additional functions are attached to a dopant molecule by coupling the properties of metal ions such as photo-response, redox properties and asymmetric catalyses. This property may be utilized to construct a bi-functional liquid crystal system.

Because of these characteristics, the ΔΛ-isomerism of transition metal *tris*-chelates bears unique steric and electronic characters, not achievable by carbon centers, and they are expected to provide significant new insights into the understanding of chiral liquid crystal phases [32–34].

In the present review, we would like to show how the above characteristic properties are manifested by using a chiral metal complex as a dopant in liquid crystal phases. The works are selected to focus on the following two issues: the first topic is the mechanism of induction of the helical arrangement involving tens of thousands of host molecules by a single chiral molecule. What is a factor determining helical sense of such mesoscopic arrangement? In relation to this, we also discuss what property of metal complex is a main factor to attain high HTP. This point is, of course, of practical importance to develop an efficient dopant [24–28]. As the second issue, we attempted to develop a bi-functional molecule such as a light responsive dopant [28–31].

## 2. Application of β-Diketonato Complexes as Chiral Dopants

The first attempt of using metal complexes as a dopant was reported by Spada and coworkers [32]. They demonstrated that the enantiomerically pure metal acetylacetonates, [M(acac)_3_], which belong to the *D*_3_ point group, exhibited high ability to induce chiral nematic phases. Hoshino *et al.* reported the Δ-enantiomer of a Ru(II) complex [Ru(acac)_2_L] (L = an elongated mesogenic derivative of bpy, 5,5’-(4-octylphenyloxycarbonyl)−2,2’-bipyridyl). In contrast to [M(acac)_3_], the complex was designed to have *C*_2_ symmetry with two acetylacetonato moieties acting as “chiral blades” [33]. This type of metal complex was found to exhibit a remarkably high helical twisting power (HTP) for nematic liquid crystals. As the attempts of using other kinds of metal ions, Shirakawa *et al*. reported the binol-derived titanate dopants [35]. The very high HTP values are also reported using the metal complexes of Zn(II) and Ti(IV) [36–38].

**Scheme 1.**
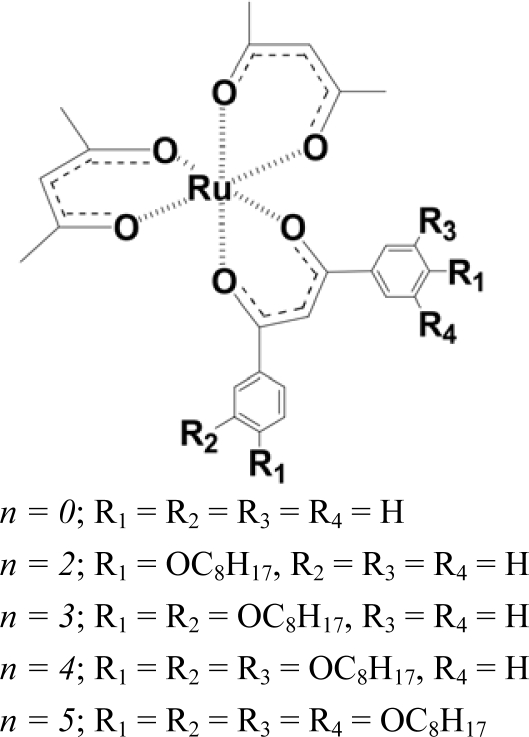
Structure of the Complexes (Illustrated for the Δ-Enantiomers) (modified from reference [24]).

Motivated by this successful approach, we elaborated the molecular design in order to improve the dopant performance [24]. As a result, a new series of Ru(III) complexes [Ru(acac)_2_(L-*n*)] (L-*n* = a dibenzoylmethanate ligand substituted with *n* octyloxy chains), were prepared (Scheme 1) and optically resolved by means of a clay column chromatography [24,39]. For example, the number of octyloxy groups (*n*) was varied from 0 (unsubstituted) and 2 through 5 by a systematic synthetic approach. The induced CD (ICD) spectra were measured for MBBA materials doped with either Δ- or Λ- enantiomers at various concentrations. Table 1 summarizes HTP values at room temperature in MBBA.

## 3. Molecular Mechanism in Induction of Chiral Nematic Phases

**Figure 1.**
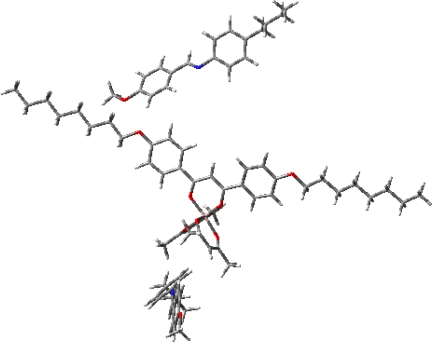
The energy-minimized structure of [Δ-[Al(acac)_2_(L-*2*) + 2MBBA], showing the mutually left-handed orientation of two MBBA molecules. This figure is a modified version of the one from reference [24].

Theoretical interpretation for the structure-HTP correlation as shown in Table 1 may require some knowledge of the chirality and orientational characteristics of the dopant used. A computational approach to HTP based on the atomistic details is usually taken to determine the equilibrium structure of a dopant [12]. Thus we conducted *ab initio* calculations for the most stable geometry of an Al(III) complex with n = 2. There were a number of conformers encountered that must be carefully sorted out before meaningful chirality parameters could be enumerated. We therefore resorted to further calculations of the energy minimized configuration of a trio of the complex and a pair of MBBA molecules [Δ-Al(acac)_2_(L-*2*) + 2MBBA], and investigating the chirality recognition in this locus. Due to the presence of such ligands as L-*n*, we could conclude that our dopants will align generally like rodlike mesogens with the biaxiality increasing with *n* [24,25] Figure 1 shows the angled view of the energy-minimized structure of [Δ-Al(acac)_2_(L-*2*) + 2MBBA]. It is noted that the mutually left-handed orientation of two MBBA molecules is achieved, when these host molecules are intervened between two acac ligands. This is completely in accord with the experimental observation that the Δ-enantimer induced the left-handed helical arrangement in a chiral nematic phase. In other words, an elongated ligand helped a dopant molecule to align in a host medium, while two acac ligands achieved the helical twisting of the host molecules that were in contact with the dopant. It was suspected that such local helicity would propagate through a liquid crystal medium to construct a helical arrangement on a micrometer scale. As for this propagation process, we also conducted further theoretical investigation based on a continuum medium model that the local twisting could be an origin of macromolecular helical architectures [25]. According to the model, the host molecules interacted attractively with dopant, being twisted in the same direction as the helical conformation of the ligands. Interaction energy was assessed as a function of the dihedral angle between the two host molecules, leading to a quadratic dependence with a minimum at the equilibrium twisting angle. Based in this, we derived the expression, in which helical twisting power was given in terms of the equilibrium twisting angle of a pair of strongly interaction host molecules.

## 4. Control of Handedness in Chiral Nematic Phases on the Basis of Molecular Design

**Figure 2.**
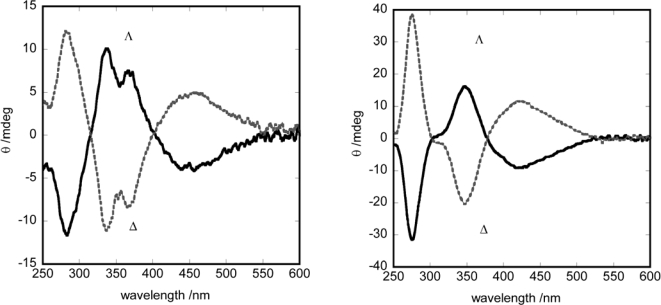
CD spectra of enantiomers in methanol (left) [Ru(acac)_2_L_per_C_6_] (right) [Ru(acac)_2_L_para_].

**Scheme 2.**
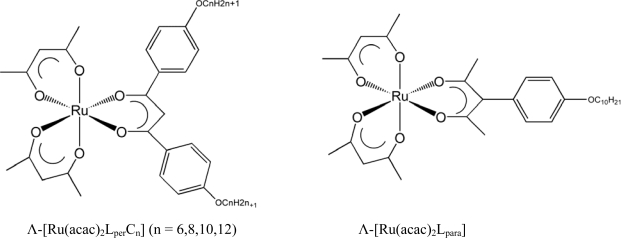
Structures of the Complexes Studied (Illustrated for the Λ-Enantiomers). This figure is modified one from reference [27].

As a consequence of the mechanism of helical induction as explained above, we postulated a model in which that the relation between ΔΛ configuration and the helical sense would depend crucially on the molecular orientation. Based on this prediction, two types of tris(chelated) molecules were compared as a chiral dopant, as shown in Scheme 2. One was elongated in the direction perpendicular to the C_2_ axis (denoted by [Ru(acac)_2_L_per_C_n_] (n = 6,8,10,12)), while the other was elongated in the direction parallel with the C_2_ axis (denoted by [Ru(acac)_2_L_para_]). These two molecules were expected to orient with their C_2_ axes perpendicularly to or in parallel with the director of a host, respectively. Their optical purity was ascertained by the circular dichroism spectra of their methanol solutions in Figure 1. When these complexes were dissolved as a dopant in a nematic host (EBBA), they showed the induced circular dichroism (ICD) as shown in Figure 2. Such intense ICD spectra of positive and negative sings of CD for [Ru(acac)_2_L_per_C_n_] and [Ru(acac)_2_L_para_] meant the induction of right(*P-*) and left handed (*M-*) helix, respectively. The chirality versus twist sense correlation for complex 1 was therefore concluded to be Δ-*M* and Λ-*P* and for complex 2 Δ-*P* and Λ-*M*, respectively. In other words, the Λ-isomer of [Ru(acac)_2_L_per_C_n_] induced right-handed helix, while the Λ-isomer of [Ru(acac)_2_L_para_] induced left-handed helix as summarized in Table 2 in terms of HTP values [27]. These examples demonstrate that the opposite handedness of induced helices for the complexes with the same Λ-configuration arises from the distinction of the elongation direction between L_per_ and L_para_.

Extending the above works, and also with the purpose of clarifying the mechanisms of helical induction, the order parameters (*S*) of dissolved metal complexes were determined by means of polarized UV-vis measurements on nematic liquid crystals doped with racemic Co(III) complexes [28]. A tris(*β*-diketonato)complex, [M(acac)_2_(LC_12_)] (Co(III); LC_12_ = 1,3-didodecyloxyphenyl-1,3-propanedionato), was synthesized. Here LC_12_ was designed to be elongated perpendicular to the molecular *C*_2_ axis. The enantiomers were dissolved as chiral dopants in three kinds of nematic liquid crystals, *N*-methoxybenzylidene-4-*n*-butylaniline (MBBA), *N*-ethoxybenzylidene-4-*n*-butylaniline (EBBA), and a mixture of 4-(4-alkylcyclohexyl)benzonitrile and 4-(4-alkylcyclohexyl)-4’-cyano-biphenyl derivatives (ZLI-1132). The sign of HTP was determined by measuring the induced circular dichroism (ICD) spectra in the range of 350∼400 nm. The positive or negative ICD spectrum was related to the formation of *P*-(right-handed) or *M*-(left-handed) helix, respectively. The results of HTP measurements are summarized in Table 3. The direction of the elongated ligand in [Co(acac)_2_(LC12)] was determined from the anisotropic ratio of their polarized electronic absorption spectra in the doped state. It was found that the long axis of the ligand, LC12, really aligned in the direction of the director with *S* = 0.5 ± 0.05. These results supported the induction mechanisms of a chiral metal complex as theoretically proposed in the preceding section.

## 5. Design of Photoresponsive Dopant of ΔΛ-Isomerism

As a host of photoresponsive species, liquid crystals are quite interesting in comparison to ordinary liquid and crystal media [14–23]. In particular, the chiral nematic (N*) phases, which are characterized by the superstructures with a helical arrangement, may undergo macroscopic structural transformations due to the photo-induced reactions of dissolved species. A number of attempts based on this strategy have been reported by using photoresponsive chiral organic and polymer compounds [14–23]. In contrast, there are only a limited number of works reporting the use of chiral metal complexes for those purposes [40–42]. Horie *et al.*, for example, studied the photoracemization of tris(β-diketonato)chromium(III) complex dissolved in a liquid crystal. The asymmetric synthesis was also attempted by illuminating a racemic mixture with a circularly polarized light [40,41].

**Scheme 3.**
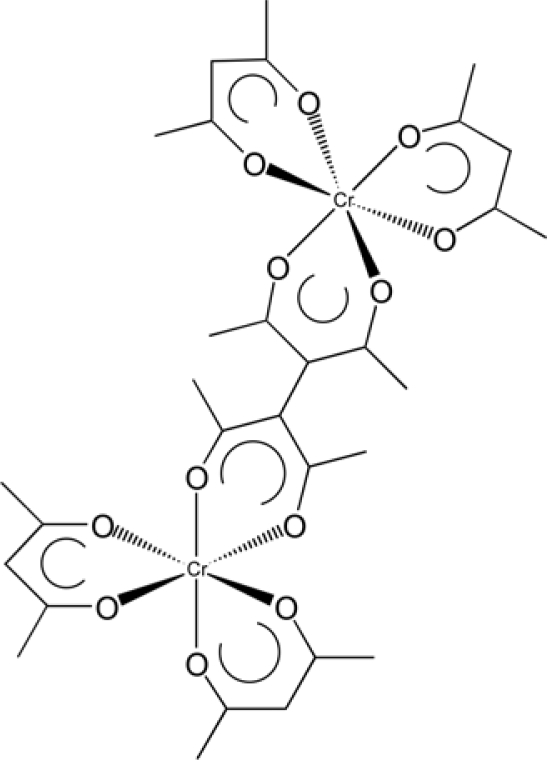
Structure of ΔΔ-[Cr(acac)_2_(taet)Cr(acac)_2_]. Modified from reference [26].

We studied the photo-epimerization of the chiral linkage effect of a binuclear acetylacetonato chromium(III), ΔΔ-[Cr(acac)_2_(taet)Cr(acac)_2_] (Scheme 3), by dissolving it in liquid crystal phases [29]. The linked compounds were synthesized by the thermal reaction between [Cr(acac)_3_] and a bridging ligand, tetraacetylmethane (taetH_2_) (Scheme 3). The diastereomeric separation of these oligomers was performed by chromatographic resolution on a chiral column [39]. The HTP of Cr(III) oligomers are summarized in Table 4, indicating the linkage effects. The table also includes the results for monomeric complexes ([M(acac)_3_]) [32]. According to the table, the enhancement of helical twisting power was not realized by connecting two monomer units with the bridging ligand, taet. Based on the present model, one reason for this lies in the fact that two connected units are perpendicularly twisted with respect to their C_2_ axes.

In order to investigate the photo-responsive properties of these liquid crystal systems, a UV light (335 nm) was irradiated at room temperature onto a glass cell containing ZLI-1132 doped with ΔΔ -[Cr(acac)_2_(taet)Cr(acac)_2_]. The change of helical pitch length of the host medium was caused by the photoisomerization of the Cr(III) complex as a dopant. The degree of photoisomerization was determined from the circular dichroism spectra of an isotropic phase of the hosts at higher temperature. The first order rate constant (k_r_) was obtained from the initial slope of the CD absorption. In a chiral nematic phase, the quantum yield was obtained to be 7.9 × 10^−4^, which was 30% of that in a homogeneous solution. This was an example that a photo-responsive metal complex behaved in a different way between a homogeneous solution and a liquid crystal host.

**Scheme 5.**
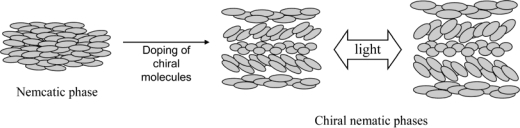
Orientation control of liquid crystals under illumination. This is modified one from reference [26].

We also pursued the possibility to control the helical pitch of a chiral nematic liquid crystal by use of a photo-responsive metal complex [30]. The principle of our attempt is shown by the schematic drawing in Scheme 5.

**Scheme 6.**
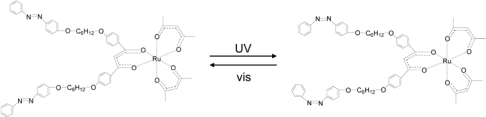
Isomerization of photoresponsive chiral Ru(III) complex, [Ru(acac)_2_(L_azo_)_per_], where acac and cis/trans photoisomerization in methanol by illuminating UV or visible light. Modified from reference [30].

In order to elucidate such a photo-responsive system based on metal complexes, a novel Ru(III) complex, [Ru(acac)_2_(L)], was synthesized, where acac and L denote acetylacetonato and 1,3-bis-{4-[6-(4-phenylazo- phenoxy)-hexyloxy]-phenyl}-propane-1,3-dione, respectively (Ru(acac)_2_(L_azo_)_per_). It should be noted that this complex undergoes a cis/trans photoisomerization in methanol solution by illuminating with UV (360 nm) or visible (450 nm) light (Scheme 6). When the complex was doped into a room temperature nematic liquid crystal (ZLI-1132), it induced a chiral nematic phase. Under the illumination of UV or visible light, the helical pitch of a chiral nematic phase changed reversibly by the amount of 50%. In corresponding to the change, the helical twisting power (HTP) of the complex varied as shown in Table 5 at 35 °C. As far as our literature survey was concerned, this was a first attempt of combining ΔΛ chirality and cis/trans isomerization for the development of a photoresponsive dopant.

**Scheme 7.**
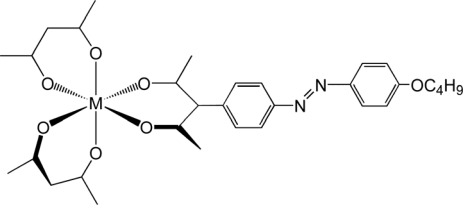
Δ-[M(acac)_2_(L_azo_)_para_] (trans form) modified from reference [28].

**Scheme 8.**
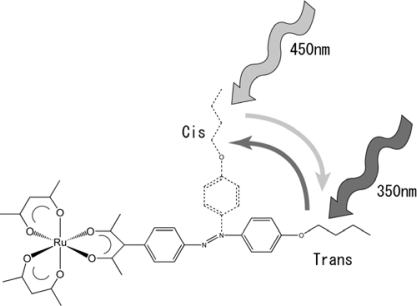
Photoresponsive behavior of [Ru(acac)_2_(L_azo_)_para_].

The photoresponsive behaviour of [Ru(acac)_2_(L_azo_)_para_] and [Co(acac)_2_(L_azo_)_para_] was also studied as shown in Scheme 7 [28]. In case of the chloroform solution of [Ru(acac)_2_(L_azo_)_para_], the absorption peak at 356 nm decreased under the illumination of a UV light shorter than 400 nm. The absorption intensity of the peak recovered reversibly when the solution was illuminated by a visible light longer than 400 nm. The results were ascribed to the cis-trans isomerization of the azobenzene moiety in [Ru(acac)_2_(L_azo_)_para_]. The similar change was also observed for [Co(acac)_2_(L_azo_)_para_]. No change was induced in CD spectra under the irradiation of either UV or visible light. This was rationalized in terms of the assumption that the isomerization of the azobenzene moiety occurred in the plane perpendicular to the vicinal acac- ligand. The similar reaction was studied by doping these complexes into a nematic host. The reversible change of HTP was observed when Δ- or Λ-[M(acac)_2_(L_azo_)_para_] doped in a ZLI-1132 sample was photo-irradiated. HTP (μm^−1^) varied from 27 to 16 for the Δ-isomer and from −31 to −18 for the Λ-isomer for Ru (III), respectively. The results could be explained by the change of order parameter *S* before and after photo-irradiation. The cis-type of L_azo_ in [M(acac)_2_(cis-L_azo_)] (M = Co(III) and Ru(III)) was assumed to align no longer with the C_2_ axis, leading to the decrease of S value. These situations are schematically shown in Scheme 8.

## 6. Application of New Spectroscopic Method to Observation of Structural Changes

**Scheme 9.**
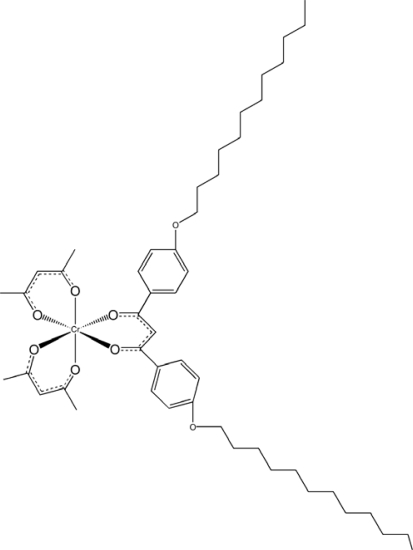
Structures of Λ-[Cr(acac)_2_(2C12)] (acac = acetylacetonate; 2C12 = 4,4’-didodecyloxyated dibenzoylmethanate).

**Scheme 10.**
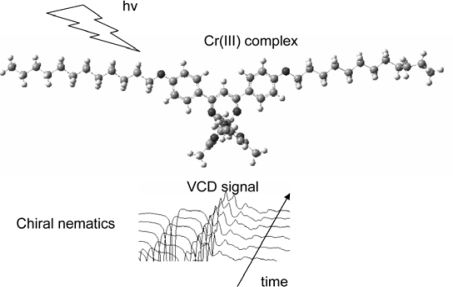
Real time monitoring of helical pitch modulation by means of vibrational circular dichroism (VCD) spectroscopy. Modified from reference [26].

A number of spectroscopic methods are applied for observation of structural changes in liquid crystal phases such as light scattering, NMR and electronic circular dichroism spectra [4]. Recently vibrational circular dichroism (VCD) spectroscopy has been applied to chiral liquid crystals for photoirradiation [31,43]. We attempted the real time monitoring of helical rewind process in chiral nematics liquid crystals by use of VCD spectroscopy [31]. VCD was applied for monitoring *in situ* the rewind of supramolecular helices in a chiral nematic phase under the illumination of UV light (365 nm). Here the change was caused by the photoracemization of a doped chromium(III) complex. For that purpose, a novel complex, [Cr(acac)_2_(2C12)] (acac = acetylacetonate; 2C12 = 4,4’-didodecyloxyated dibenzoylmethanate), was synthesized (Scheme 9). It should be noted that the rewinding process of the helix was visualized as the change of the spectral shape at each of the four vibrational peaks (Scheme 10). This was the first application of VCD spectroscopy to photoresponsive systems.

## 7. Conclusions

This review article summarizes our recent attempts at using the ΔΛ isomerism as a chiral dopant inducing chiral nematic phases. As a result, a series of metal complexes expressed by the formula of [M(III)(blade)_2_(backbone)] (M = Ru, Co, Cr) were shown to have extremely high HTP values. The backbone ligands provided an essential factor for molecular orientation to achieve high HTP in addition to the ΔΛ- chirality. The bi-functional nature of metal complexes, particularly of transition metal complexes, was coupled with this doping effect. For example, photoresponsive dopants were designed with the linkage effects of Cr(III) polynuclear metal complexes. Moreover we attempted the combination of the ΔΛ chirality with cis-trans isomerization of an azobenzene group for the photomodulation of chiral nematics. Finally VCD (vibrational circular dichroism) spectroscopy was proposed as a novel spectroscopic method to monitor the change of helical pitch *in situ*.

## Figures and Tables

**Figure 3.**
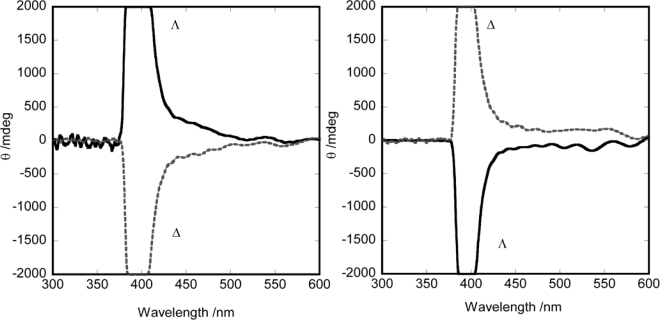
ICD spectra for EBBA doped Δ- and Λ-enantiomers. (left) [Ru(acac)_2_L_per_C_12_] (right) Λ-[Ru(acac)_2_L_para_].

**Table 1.** Values for HTP (β_M_)^a^ at room temperature of [Ru(acac)_2_(L-*n*)] in MBBA.

***n***	***β*_M_/μm^−1^**	
	Λ	Δ
0	−	−48
2	176	−175
3	131	−133
4	(84)	−95
5	70	(−38)

a The signs + and − indicate that right- and left-handed helical superstructures are induced, respectively. The values were evaluated according to Equation 1. – Not determined. The parentheses are estimated as one sample so that they were considered to contain a large error. This table is modified from one in reference [24].

**Table 2.** Values for HTP (*β*_M_) at room temperature of [Ru(acac)_2_L_per_C_n_] and [Ru(acac)_2_L_para_] in MBBA. This table is modified one from reference [27].

**(a)** [Ru(acac)_2_L_per_C_n_]				
***β*M/μm−^1^**	**n = 6**	**8**	**10**	**12**
Λ	130	176	130	120
Δ	−146	−175	−140	−127

(b) [Ru(acac)_2_L_para_]	
***β*M/μm^−1^**	**n = 10**
Λ	−24
Δ	24

* The signs + and – indeicate that *P-* and *M-* helical superstructures are induced respectively.

**Table 3.** Values for HTP (*β_M_* /μm^−1^) of Δ, Λ-[M(acac)_2_(LC_12_)] in MBBA (30 °C), EBBA (60 °C) and ZLI-1132 (35 °C). This table is modified from one in reference [28].

**Dopant**	**MBBA**	**EBBA**	**ZLI-1132**
Δ-Ru(acac)_2_(LC_12_)]	−127	−66	−55
Λ-Ru(acac)_2_(LC_12_)]	120	72	58
Δ-[Co(acac)_2_(LC_12_)]	−144	−64	−69
Λ-[Co(acac)_2_(LC_12_)]	135	68	71

**Table 4.** The spectroscopic properties of [Cr(acac)_3_] oligomers and their HTP values in MBBA and ZLI-1132 at 30 °C. Table modified from reference [29].

**Oligomer**	**Isomer**	**β(μm^−1^)/MBBA**	**β(μm^−1^)/ ZLI-1132**
Monomer	Λ	+99.5	+23.0
	Δ	−91.0	−25.3
Binuclear species	ΛΛ	+97.9	+26.0
	ΔΔ	−88.9	−29.1
Trinuclear species	ΛΛΔ or ΛΔΛ	+128.0	−
	ΔΔΛ or ΔΛΔ	−90.9	−

**Table 5.** Photo-modulation of HTP in 0.2% solutions of enantiomeric [Ru(acac)_2_(L_azo_)_per_] doped in ZLI-1132 at 35.0 °C. The experiments were performed for one sample so that they might contain a large error (this table is a modified version of one in reference [30]).

		**Initial**	**Visible**	**UV**
Λ	*β*_*M*_ /μm^−1^	+38	+34	+22
Δ	*β_M_* /μm^−1^	−50	−44	−27
